# Relationship between perceived teacher support and learning engagement among adolescents: Mediation role of technology acceptance and learning motivation

**DOI:** 10.3389/fpsyg.2022.992464

**Published:** 2022-09-30

**Authors:** Fuhai An, Jingyi Yu, Linjin Xi

**Affiliations:** Jing Hengyi School of Education, Hangzhou Normal University, Hangzhou, China

**Keywords:** perceived teacher support, learning engagement, technology acceptance, learning motivation, mediation

## Abstract

This study is aimed at investigating the relationship between perceived teacher support and learning engagement and exploring the mediation role played by technology acceptance and learning motivation. It adopted a structural equation modeling (SEM) approach, with sampling 467 students from four middle schools in eastern China. The research findings showed that perceived teacher support is significantly associated with learning engagement. Learning motivation plays a mediating role in the relationship between perceived teacher support and learning engagement. There is the chain mediating effect of technology acceptance and learning motivation on the relationship between perceived teacher support and learning engagement. All of these are of great importance for the teachers in the middle schools, as they help to increase students’ engagement with learning activities considering the background of the deep integration of information technology and education teaching.

## Introduction

With the rapid development of information technology, digital curriculum resources nowadays play a crucial role in teaching-learning process. With the easy access to the Internet, learning relying on smart terminals has become an important way for students to engage in learning activities (Jang et al., 2021). With the background of deep integration of technology and education teaching, how to improve students’ learning engagement and identify the potential influencing factors have also become a hot spot for research (Zhang, 2012). Learning engagement is a continuous act of learning activity, accompanied by a state of passionate emotion (Skinner and Belmont, 1993). It is an effective indicator of students’ progress toward achieving desired academic and social outcomes (Henrie et al., 2015), as well as has a significant impact on academic achievement (Jian, 2022). Previous studies have shown that learning engagement can be influenced by factors such as teacher support (Liu et al., 2018) and learning motivation (Yin and Wang, 2016). Social support theory and self-determination theory suggest that external supportive behaviors have a direct impact on a person’s motivation formation and sustained engagement (Hilkevitch, 1977; Deci and Ryan, 1985). By exploring the factors that influence students’ learning engagement, teacher support was found to be a key factor, while perceived teacher support was associated with learning engagement (Liu et al., 2018). Although many studies have shown that perceived teacher support affects students’ learning engagement (Wang et al., 2017), following the integration of information technology into education and teaching, does perceived teacher support still affect students’ learning engagement in teaching and learning environments involving technology? Previously-reported research lacks in-depth theoretical exploration and scientific findings in this area. More importantly, learning with the help of smart devices (such as computers and iPad) is now becoming an important part of education and the trend seems irreversible. Faced with the new environment and equipment, middle school students also have a lot of problems in terms of learning engagement and motivation. Part of the issue can be solved with the guidance of the teachers. However, the teachers themselves do not have all the answers and the scientific guidance for them is also urgently needed. Therefore, we conducted this study with middle school students as the main target. Meanwhile, very few research focus on whether technology acceptance and learning motivation play a chain mediation role between perceived teacher support and learning engagement.

### The relationship between perceived teacher support and learning engagement

It has been demonstrated that there is a correlation between perceived teacher support and learning engagement (Yang et al., 2021). Perceived teacher support is regarded as students’ perceptions of teachers’ attitudes and behaviors toward their studies and daily lives (Babad, 1990). It includes several dimensions, including academic support and emotional support (Johnson et al., 1985; Patrick et al., 2007). More specifically, teacher academic support explains students’ view of what the instructor cares and how much the students have learned, while teacher emotional support indicates students’ sense of the teacher’s care about the students as individuals (Johnson et al., 1985). As a contextual factor, influences from teachers were framed within several theoretical frameworks, e.g., social support theory (Hilkevitch, 1977), and self-determination theory (Deci and Ryan, 1985). Based on social support theory, individuals perceive supportive behaviors from their social network as universally gainful and contributing to their psychological health and development (Berkman and Syme, 1979). According to self-determination theory, the external environment can enhance their internal motivation, promote internalization of external motivation, and sustain engagement by meeting three basic psychological needs: autonomy, competence, and belonging (Deci and Ryan, 1985). Overall, teacher support, as a form of social support, is likely to influence students’ learning engagement. Researches have reported a direct link between perceived teacher support and learning engagement, teacher support can contribute to students’ learning engagement (Roorda et al., 2017; Strati et al., 2017). In Wentzel’s study, perceived teacher support can facilitate students’ willingness to cognitively and behaviorally engage in academic tasks, teacher support was positively related to students’ interest in engaging in interactive classroom tasks (Wentzel, 1997). Another research found that teacher support may generate good teacher-student connections that can improve students’ social interaction and intellectual skills within a classroom environment (Huang et al., 2022). Positive relationships between teachers and students can be a key source of supporting students’ academic efforts and, motivating them to engage more in academic activities and learning tasks (Cooper, 2014). As evidenced by these researches, teacher support can lead to improved students’ learning engagement.

### The mediating role of technology acceptance

It has been demonstrated that perceived teacher support can increase students’ technology acceptance (Lai, 2015). According to Davis’s technology acceptance model (TAM), perceived usefulness and perceived ease of use are antecedent variables that influence users’ attitudes toward information technology (Davis, 1989). The two factors affect the actual use of behavior directly and indirectly (Ajzen, 1991; Teo and van Schaik, 2012). Venkatesh and Davis (2000) introduced a series of social influence variables (Subjective Norm, Voluntariness, Image, and Experience) into the classical TAM model in 2000, censored the effect of attitude on willingness, and proposed TAM 2. The comprehensive model TAM 3, introduced by Venkatesh et al. in 2008, includes perceived usefulness and perceived ease of use. It highlights that community influence, system characteristics, individual differences, and convenience are influential in perceived usefulness and perceived ease of use. The UTAUT theory suggests that willingness and convenience to use technology contributes to the actual use of behavior (Venkatesh et al., 2012). Among them, perceived ease of use and perceived usefulness are the primary factors. Perceived usefulness refers to the degree to which an individual feels technology that help them to complete a given work efficiently and productively (Davis, 1989). In contrast, perceived ease of use indicates the degree to which a person feels that the use of technology would be relatively free of effort. In the field of education, technology acceptance is considered as a precondition for learners to utilize information technology for the improvement of learning (Hsieh et al., 2017). As shown in recent studies, external variables (e.g., teacher support) influence learners’ perceived ease of use and perceived usefulness of technology (Bai et al., 2019). A research reported that teacher support affects students’ perceived usefulness and ease of use with technology (Lai et al., 2012). In another study, when students feel more teacher support in technology-supported learning environments, they find technology more helpful for learning and easier to use (Wang et al., 2021).

### The mediating role of learning motivation

The evidence shows that there is a correlation between learning motivation and students’ learning engagement (Wu et al., 2020). Learning motivation is the total of the incentives that positively compel the choosing of a given activity or goal (Jarvis, 2005). According to Deci and Ryan’s self-determination theory (SDT) (2000), the ego has a dynamic role in the motivational process, the individual is free to choose his or her own motivation on the basis of the environment and needs. A positive environment and constant satisfaction of needs will further motivate the motivation. Learning motivation attributes to favorable academic achievements as well as students’ perceptions of pleasure, happiness, and satisfaction, which in turn further encourage their commitment to learning (Dishon-Berkovits, 2014). For example, Barba et al. (2016) found that students’ learning motivation has the positive effect on their learning interest, learning engagement and performance. Ecosystem theory highlights that environmental factors generally act through a number of intrinsic individual variables (Bronfenbrenner, 1979). Social support received or perceived by an individual is more likely to affect individual behavior by the work of some intrinsic factors (e.g., learning motivation) (Helgeson and Lopez, 2010). In other words, teacher support can have significant impact on students’ learning achievement by facilitating their learning motivation. The study’s results showed that students with greater awareness of teacher support were more motivated to learn in the classroom and to achieve better academic outcomes (Ruzek et al., 2016). In contrast, students with less teacher support are more focused on avoiding criticism, leading to serious damage to learning efficiency and academic achievement (Deci and Ryan, 2000). Another study also showed that learning motivation was positively linked to both teacher feedback and learning engagement in a technology-supported instructional environment (Pan and Shao, 2020). In other words, the more feedback and support teachers offer in a technology-supported educational setting, the more motivated students are to study and the more time and energy they dedicate to learning.

### The relationship of technology acceptance and learning motivation

Some evidence suggests that technology acceptance has a more satisfactory influence on students’ learning motivation in technology-involved educational contexts (Zuo et al., 2022). Based on Venkatesh et al.’s integrated model TAM 3 (2008), external variables, by influencing perceived usefulness and perceived ease of use, can to some extent influence users’ behavioral intentions and thus their behavior in using the technology (e.g., attitudes toward use, frequency of use). Self-determination theory underlines that situational factors (e.g., instructor behavior, social interactions) can influence learners’ learning status (e.g., learning engagement) by enhancing or inhibiting learners’ learning motivation (Deci and Ryan, 1985).

Combining the technology acceptance model and the self-determination theory, students’ motivations to study and learning engagement increase when they consider technology to be more useful, and easy to use (Horzum et al., 2015). However, some studies have found that using technology for learning can also be a challenging task for students (Mtebe and Raisamo, 2014). If the technology is difficult, students will need to spend time and effort in learning and use it, which can lead to a decrease in their willingness to use it (Tick, 2019). As a result, students’ learning motivation and learning engagement would be hindered. Therefore, it is crucial to investigate relationships between technology acceptance, learning motivation and learning engagement and to explore the ways of improving students’ technology acceptance to enhance their learning motivation and learning engagement.

### Aims and hypotheses

**FIGURE 1:**
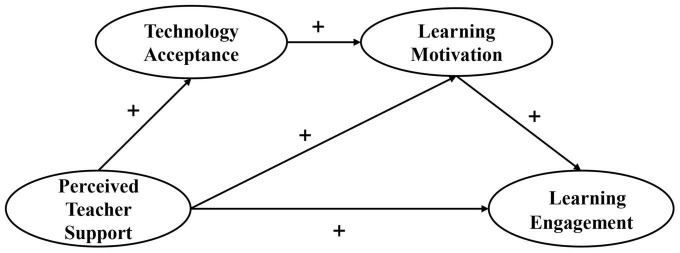
Hypothesized research model.

It has been shown that students’ perceived teacher support was associated with technology acceptance, learning motivation, and learning engagement (Yıldırım, 2012; Lai, 2015; Ansong et al., 2017). However, to the best of our knowledge, very few study has investigated whether technology acceptance and learning motivation can act simultaneously as mediators between perceived teacher support and learning engagement. In addition, following the deep integration of information technology into teaching and education, Chinese teachers are still somewhat confused about how to improve middle school students’ learning engagement. There is a need of scientific guidance on the subject. This study aims to examine the relationship between students’ perceived teacher support and learning engagement through the mediation of technology acceptance and learning motivation. It will provide a valuable reference for middle school teachers and researchers to improve students’ learning engagement under the background of the deep integration of information technology into teaching and education. We provide a hypothetical model based on the literature review discussed above (Figure 1). The hypotheses of this research are as follows:

Hypothesis 1 (H1): Perceived teacher support is directly and positively associated with learning engagement.

Hypothesis 2 (H2): Learning motivation mediates the relationship between perceived teacher support and learning engagement.

Hypothesis 3 (H3): Technology acceptance and learning motivation show a chain-mediating effect in the relationship between perceived teacher support and learning engagement.

## Materials and methods

### Participants and procedures

This study adopted random sampling method to select four middle schools located in Hangzhou, Zhejiang Province, East China. Firstly, considering that there are some differences in education between urban and rural areas in China, we randomly selected two middle schools each from urban areas and rural areas (according to the standard of administrative region division in China). Secondly, we conducted a stratified sampling according to grade levels. Considering that 9th graders are busy preparing for their midterm exams and may not pay attention to completing the questionnaire, we only surveyed 7th and 8th graders in four selected schools. Finally, we used whole group sampling and took the class as the unit. Two classes were randomly selected from each of grades 7 and 8, and all students in the selected classes were surveyed.

According to the calculation formula of sample size *N* = *Z*^2^*[P*(1-*P*)/*E*^2^], we set the confidence level to 95% (*Z* = 1.96), error value *E* = 5%, probability value *P* = 0.5, and calculate the sample size *N* = 384, so our sample size should be above 384. In addition, Barrett argued that SEM generally uses the built-in maximum likelihood method, which severely inflates chi-square values at sample sizes greater than 500. This can lead to a poor fit of the model (Barrett, 2007). Therefore, we controlled the sample size between 384 and 500. In order to ensure the comprehensiveness, scientificity and representativeness of the survey, and in consideration of the fact that there may be invalid questionnaires in the actual survey, we surveyed a total of 501 students and organized the collected data, leaving 467 data after eliminating invalid data (93.2% effective rate).

The characteristics of the participants are shown in Table 1. Of the 467 samples, 50.1% (*N* = 234) were male, and 49.9% (*N* = 233) were female. 12.4% (*N* = 58) were 12°years old, 56.5% (*N* = 264) were 13°years old, 28.1% (*N* = 131) were 14°years old, 3% (*N* = 14) were 15°years old. 52.7% (*N* = 246) were from Grade Seven, and 47.3% (*N* = 221) were from Grade Eight. 25.3% (*N* = 118) were from Urban School 1, 26.3% (*N* = 123) were from Urban School 2, 23.6% (*N* = 110) were from Rural School 1, and 24.8% (*N* = 116) were from Rural School 2.

**TABLE 1:** Demographic statistics (*N* = 467).

Variables	Frequency	Percent (%)
**Gender**		
Male	234	50.1
Female	233	49.9
**Age group**		
12°years old	58	12.4
13°years old	264	56.5
14°years old	131	28.1
15°years old	14	3.0
**Grade**		
Grade 7	246	52.7
Grade 8	221	47.3
**Types of schools**		
Urban school 1	118	25.3
Urban school 2	123	26.3
Rural school 1	110	23.6
Rural school 2	116	24.8

This research was part of a project aimed to evaluate the influence of non-intellectual characteristics on learning situation, and this study was initially authorized by the Ethics Committee of Hangzhou Normal University and the administrative department for chosen school. The research took place over 12°days in the midst of a normal academic semester, and professional administrators monitored it. The students who accepted to participate in the research were led to a classroom where a computer was provided with the questionnaire. Participants were invited to reply as frankly as they could, and were given assurance that their comments would remain anonymous. They took roughly 30 min to fill out the form.

### Measuring instrument

#### Teacher support

Perceived teacher support was measured using the Perceived Teacher Support Scale (Patrick et al., 2007). The scale is a condensed version of the scale created by Ryan and Patrick (2001) and modified by Patrick et al. (2007). The Scale includes the Teacher Academic Support Scale (four items, e.g., “*My teacher cares about my learning*.”) and the Teacher Emotional Support Scale (four items, e.g., “*My teacher understands how I feel about things*.”), which have been proved to be reliable and valid in previous studies (Patrick et al., 2007). The participants were requested to react to the statements on a five-point scale from 1 (completely disagree) to 5 (completely agree) depending on their felt support from teachers. Thus, as an indicator of perceived teacher support, higher scores indicate higher levels of perceived teacher support. The Cronbach’s alpha value in this study was 0.878.

#### Technology acceptance

Technical acceptance was measured using the Technical Acceptance Scale. The scale was compiled by Teo (2009), and adapted from TAM technology acceptance model created by Davis in 1989. The TAM has been extensively validated in the Chinese context, showing adequate concurrent and construct validity. The scale consists of four dimensions with 12 items: perceived usefulness (three items, e.g., “*Use of technology helps expand learning opportunities.*”) perceived ease of use (three items, e.g., “*Using technology does not require many instructions*.”), attitudes toward technology use (three items, e.g., “*Computers make work more interesting.*”) and behavioral intentions (three items, e.g., “*I will use computers in the future.*”). The participants were requested to react to the statements on a 5-point scale from 1 (completely disagree) to 5 (completely agree). Thus, as an indicator of technology acceptance, higher scores indicate higher levels of technology acceptance. The Cronbach’s alpha value in this study was 0.942.

#### Learning motivation

Learning motivation was measured using the revised Learning Motivation Scale (Chi and Xin, 2006). The scale was completed by Amabile et al., 1994 in 1994 and accurately translated and revised by Chi and Xin (2006). The revised scale is adapted to Chinese students and was reported to have good reliability and validity. In this scale, learning motivation is divided into intrinsic motivation (14 items, e.g., “*I am motivated by curiosity to do many things.*”) and extrinsic motivation (16 items, e.g., “*For me, the grades I can earn are the main motivation for me to try.*”). Each item was rated on a five-point Likert scale ranging from 1 (completely disagree) to 5 (completely agree). All responses were based on general feelings. Higher scores indicate higher levels of learning motivation. In the present study, Cronbach’s alpha was 0.947.

#### Learning engagement

Learning engagement was measured using the Chinese version of the Learning Engagement Scale (Fang et al., 2008). The scale was created by Schaufeli et al. (2002) and translated and revised by Fang et al. (2008). It has been found to have good validity and reliability (Shi et al., 2013). The scale includes a vitality dimension (six items, e.g., “*I feel energized when I study*.”), dedication dimension (five items, e.g., “*I find learning to be purposeful and meaningful.*”) and the focus dimension (six items, e.g., “*When I study, I feel time flies*.”). Responses were used on a five-point Likert scale ranging from 1 (completely disagree) to 5 (completely agree), with a higher score indicating higher engagement. In this study, the Cronbach’s alpha of the overall scale was 0.937.

### Data analysis

First, we first used SPSS version 26.0 (IBM, Chicago, IL, USA) to calculate the descriptive statistics and correlations. Second, a confirmatory factor analysis (CFA) was specified to test the proposed measurement structure underlying the data. Third, Mplus 8.3 was used to examine the hypothetical model in the current study. Several fitting indices were employed to assess the overall model fit. Previous researchers (Hu and Bentler, 1999) noted that χ^2^/*df* (< 3), GFI (≥ 0.90), TLI (≥ 0.95), CFI (≥ 0.95), RMSEA (< 0.06), and SRMR (< 0.08) reflect a good fit. Fourth, bootstrap methods with robust standard errors were used to test the significance of mediating effects (Hayes, 2017). The bootstrap approach provided 95% deviation-corrected confidence intervals (CIs) for these effects using a resample of 5,000 data. The significance of the indirect effects was indicated if there were no zeros in the CIs. All statistical tests were two-tailed. In addition, we also tested an alternative model to ensure that our hypothetical model is optimal. After validation, we found no significant difference between the hypothetical model and the alternative model in terms of fit indices, etc., so we removed the alternative model.

## Results

### Descriptive statistics and correlations

Table 2 shows means, standard deviations, the relationship between study variables, gender difference and urban-rural schools difference. Cronbach’s alpha was content for all study variables (i.e., α > 0.60). In terms of relevance, perceived teacher support was significantly positively correlated with technology acceptance, learning motivation and learning engagement (*r* = 0.230, *p* < 0.05; *r* = 0.373, *p* < 0.05; *r* = 0.395, *p* < 0.05). Technology acceptance was significantly and positively correlated with learning motivation and learning engagement (*r* = 0.256, *p* < 0.05; *r* = 0.153, *p* < 0.05) and there was a significant positive correlation between learning motivation and learning engagement (*r* = 0.408, *p* < 0.05). In addition, we employ Harman’s single factor test (Podsakoff et al., 2003) to investigate the common method variance since these variables were measured using a self-reported questionnaire. The results of the unrotated exploratory factor analysis extracted 10 factors with eigenvalues greater than 1. The first factor explained 23.802% of the variance (below the critical threshold of 40%). This indicates that no serious common method biases were present in the data.

**TABLE 2:** Descriptive statistics and correlations.

Variables	1	2	3	4
1. Perceived teacher support	1			
2. Technology acceptance	0.230**	1		
3. Learning motivation	0.373**	0.256**	1	
4. Learning engagement	0.395**	0.153**	0.408**	1
Range	1–5	1–5	1–5	1–5
Mean	3.928	3.736	3.853	3.586
Standard deviation	0.710	0.897	0.514	0.852
Gender difference	−0.056	0.060	−0.056	−0.082
Urban–rural schools difference	−0.007	0.018	−0.049	−0.062

**P < 0.01.

Through the Pearson’s correlations analysis among the variables, the results showed that there was no significant difference among the four variables of perceived teacher support, technology acceptance, learning motivation and learning engagement between different genders (*r* = −0.056, *p* > 0.05; *r* = 0.060, *p* > 0.05; *r* = −0.056, *p* > 0.05; *r* = −0.082, *p* > 0.05). There was also no significant difference between these four variables in the urban schools and rural schools dimensions (*r* = −0.007, *p* > 0.05; *r* = 0.018, *p* > 0.05; *r* = −0.049, *p* > 0.05; *r* = −0.062, *p* > 0.05), which was not statistically significant.

The results of the multiple variance analysis are shown in Table 3. Students in different types of schools did not have significant differences in perceived teacher support, technology acceptance, learning motivation and learning engagement (*r* = 0.034, *p* > 0.05; *r* = 0.013, *p* > 0.05; *r* = −0.041, *p* > 0.05; *r* = −0.047, *p* > 0.05). Therefore, we did not include gender and school as covariates in the model.

**TABLE 3:** Types of schools variance analysis.

Variables	Schools			Sex		
	*r*	*F*	*P*	*r*	*F*	*P*
1. Perceived teacher support	0.034	1.508	0.212	–0.056	1.466	0.227
2. Technology acceptance	0.013	0.280	0.840	0.060	2.054	0.152
3. Learning motivation	–0.041	0.982	0.401	–0.056	1.479	0.225
4. Learning engagement	–0.047	0.677	0.567	–0.082	3.154	0.075

### Measurement and structural equation model

**FIGURE 2:**
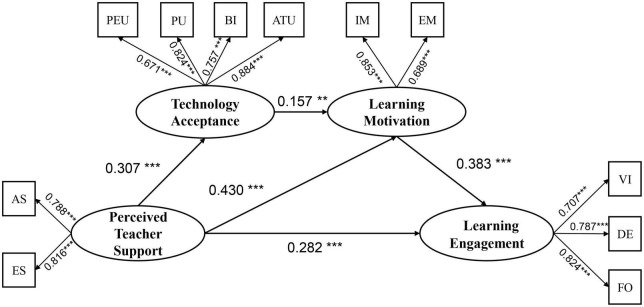
Path coefficients for the research model. PEU, perceived ease of use; PU, perceived usefulness; BI, behavioral intentions; ATU, attitudes toward technology use; IM, intrinsic motivation; EM, extrinsic motivation; AS, academic support; ES, emotional support; VI, vitality; DE, dedication; FO, focus. ***p < 0.001,**p < 0.01.

The measurement model was comprised of four potential factors and eleven indicators. Specifically, perceived teacher support had two indicators (i.e., teacher academic support and teacher emotional support); technology acceptance had four indicators (i.e., perceived usefulness, perceived ease of use, attitude toward technology use, and behavioral intention); learning motivation had two indicators (i.e., intrinsic motivation and extrinsic motivation); and learning engagement had three indicators (i.e., vitality, dedication, and focus). The results of confirmatory factor analysis (CFA) showed an acceptable fit for the measurement model [χ^2^ = 1247.659, DF = 428, CFI = 0.832, TLI = 0.832, GFI = 0.845, SRMR = 0.065, RMSEA (90% CI) = 0.064 (0.060–0.068)]. Considering the acceptable fit of the measured model, we performed SEM analysis to test the hypothesized model. The results showed that the proposed model exceeded the suggested criteria and provided a good representation of the sample relationship. The starting theoretical model (Figure 1) showed a good fit to the empirical data [χ^2^ = 136.224, DF = 60, CFI = 0.955, TLI = 0.961, GFI = 0.955, SRMR = 0.039, RMSEA (90% CI) = 0.052 (0.041–0.065)]. Figure 2 shows the standardized regression weights for this model. The results of the sample confirmed that: (1) the effect of perceived teacher support on learning engagement was significant (β = 0.282, *p* < 0.001), therefore H1 was verified; (2) the effect of perceived teacher support on technology acceptance (β = 0.307, *p* < 0.001) and learning motivation (β = 0.430, *p* < 0.001) was significant; (3) technology acceptance positively predicted learning motivation (β = 0.157, *p* < 0.05); and (4) learning motivation positively predicted learning engagement (β = 0.383, *p* < 0.001).

### Mediating effect analysis of perceived teacher support and learning engagement

Finally, we employed a bias-corrected bootstrap approach to evaluate whether the mediating effects mentioned above were significant. As shown in Table 4, (1) the indirect effect of perceived teacher support on learning engagement *via* the mediation of learning motivation was significant [effect = 0.165, 95% CI (0.087, 0.274)], 35.4% of the total effect, therefore H2 was verified; (2) the indirect effect of perceived teacher support on learning engagement *via* the chain-mediating of technology acceptance and learning motivation was significant [effect = 0.019, 95% CI (0.005, 0.046)], 4.1% of the total effect, therefore H3 was verified.

**TABLE 4:** Total, direct, and indirect effects.

Paths	Standardized estimates	95% confidence interval		Percentage of total effect	Hypothesis test
		Lower	Upper		
Total effect	0.466***	0.321	0.593		
**Direct effects**					
TS-TA	0.307***	0.166	0.433		
TS-LM	0.430***	0.292	0.568		
TS-LE	0.282***	0.116	0.446		Supported
TA-LM	0.157***	0.023	0.285		
LM-LE	0.383***	0.216	0.529		
**Indirect effect**					
TS-LM-LE	0.165***	0.087	0.274	35.4%	Supported
TS-TA-LM-LE	0.019**	0.005	0.046	4.1%	Supported

TS, teacher support; TA, technology acceptance; LM, learning motivation; LE, learning engagement. ***p < 0.001, **p < 0.01.

## Discussion

Taking middle school students in eastern China as participants, this study examined the mediating roles of technology acceptance and learning motivation between perceived teacher support and learning engagement. The research results support three proposed hypotheses: (1) perceived teacher support is directly and positively associated with learning engagement; (2) learning motivation mediates the relationship between perceived teacher support and learning engagement; and (3) technology acceptance and learning motivation show a chain-mediating effect in the relationship between perceived teacher support and learning engagement.

### Perceived teacher support and learning engagement

The current study found a significant positive correlation between perceived teacher support and learning engagement in a technology-involved instructional environment. Specifically, the more emotional and behavioral support teachers provide to students in a technology-involved instructional environment, the more energy students devote to learning. This finding is consistent with the ecosystem theory. Ecosystem theory (Bronfenbrenner, 1977) reports that, perceived teacher support, as an important component of the school microsystem, has a significant impact on students’ confidence, quality, and behavioral attitudes. Learning engagement as a behavioral attitude is influenced by the school environment, particularly perceived teacher support (Strati et al., 2017). The current findings also suggest that perceived teacher support can directly or indirectly influence learning engagement (Sadoughi and Hejazi, 2021). Teachers’ behaviors, such as providing timely feedback to students academically and emotionally caring, praising and respecting students, are significantly associated with students’ learning engagement, leading to an increase of students’ willingness to engage in learning activities (Ruzek et al., 2016). Both theoretical and empirical results suggest that perceived teacher support (both academic and emotional) is a key factor of students’ learning engagement in instructional settings involving technology (Vollet et al., 2017). However, follow the integration of information technology and educational instruction, teachers will have relatively less time for face-to-face interaction with students, which may result in teachers’ inability to provide appropriate support to students at the right time. To address this problem, teachers can give more academic and emotional support through online voice or video communication to facilitate students’ learning engagement.

### The mediating effect of learning motivation

The findings suggested that students’ learning motivation partially mediates the relationship between teacher support and students’ learning engagement. Perceived teacher support not only predicted students’ learning engagement directly but also indirectly by the mediation role of learning motivation. In other words, the higher the students’ perceived teacher support, the more motivated students are to engage in learning and more focused on learning (Ferrell, 2012; Wang and Eccles, 2013). This is because learning motivation is an important factors of influencing teaching activities and will affect learners’ learning engagement (Printrich and Schunk, 2002). According to Ryan and Deci’s self-determination theory proposed (2000), all people have three basic psychological needs of belonging, autonomy, and competence. When three basic psychological needs are met, it contributes to the formation of intrinsic motivation in students. Teachers’ supportive behaviors satisfy students’ needs for belonging, autonomy, and competence (Jin and Wang, 2019). When these needs of students are addressed, their internal and external motivation is enhanced (Deci and Ryan, 2002). This enhances learning engagement accordingly (Tseng et al., 2019). Previous research has also demonstrated that students’ motivation and learning engagement can be considerably boosted by building their relationship with their teachers (Reeve, 2012). When students are supported by their teachers, their motivation to learn is improved and they become more focused on classroom learning activities (Ryan and Patrick, 2001; Boulton et al., 2012; Lu et al., 2022). This may be related to the teacher expectation effect. According to the teacher expectation effect, the more expectations and positive emotional support teachers invest in their students, the more students are likely to develop in the directions the teachers expect (Rosenthal and Jacobson, 1968). Specifically, the more expectations and positive emotional support teachers invest in their students, the more likely they are to enhance students’ motivation and increase students’ learning engagement. Therefore, teachers should focus on the impact of academic and emotional support on students’ learning engagement. More importantly, they pay attention to motivate students in the learning process consistently by building a good teacher-student relationship, creating a democratic learning atmosphere, respecting students’ independent exploratory behaviors, and meeting their basic psychological needs.

### The mediating role of technology acceptance and learning motivation

The research results found that technology acceptance is directly and positively associated with learning motivation, as well as a chain-mediating effect in the relationship between perceived teacher support and learning engagement. According to Davis’ Technology Acceptance Model (TAM), the comprehensive model TAM3 (Venkatesh and Bala, 2008) suggests that perceived usefulness and perceived ease of use can be influenced by external circumstances (e.g., support from important people such as teachers), which can in turn influence students’ behavioral intentions. For example, perceived usefulness was found to mediate the relationship between teacher support and students’ autonomous language learning (Pan and Chen, 2021). Self-determination theory suggests that contextual factors (e.g., instructor behavior, social interactions) can enhance or inhibit learner learning motivation and be used as a basis to improve learner learning engagement (Deci and Ryan, 2000). Combining the technology acceptance model with self-determination theory gives an explanation of the relationship model. It has also been shown that, students’ perceived teacher support and higher technology acceptance are positively correlated (Lai et al., 2012), higher technology acceptance and learning motivation are related (Romero-Frías et al., 2020), higher motivation to learn promotes students’ learning engagement (Pan and Shao, 2020). The findings suggest that in a technology-involved educational setting, the more academic and emotional support students perceive from teachers, the more positive their perceptions of the usefulness and ease of use of technology, and the more motivated and engaged they are in learning when they perceive that technology is easier to use and helpful to solve problems in learning. The research findings demonstrate that teachers can improve students’ learning engagement by providing more support for them (including advice on appropriate behavior and emotional support, respect, and encouragement), giving them the impression that technology is simple to use and capable of resolving study-related issues, and encouraging them to use it. In addition, self-determination theory also emphasizes that the key factor of learning motivation is the internalization of external motivation. However, after the excessive involvement of information technology in the teaching process, the interaction between teachers and students in terms of language, emotion, and behavior becomes less and less. There are many obstacles to the development of students’ motivation without teachers’ support. Therefore, in the environment of deep integration of information technology into education and teaching, teachers should give more support to students in various aspects such as emotion and behavior, and promote the internalization of students’ extrinsic motivation and thus increase students’ learning engagement.

## Conclusion, implications, and future directions

The current study makes an innovative contribution to understanding the relationship between teacher support and learning engagement, as it emphasizes the co-mediating role of technology acceptance and learning motivation in the relationship between teacher support and learning engagement. In an instructional setting involving technology, teacher support can enhance students’ learning engagement by promoting their technology acceptance and learning motivation. In this study, the theoretical aspects of the relationship between teacher support and learning engagement in an environment of integrating information technology with educational teaching were explored in depth and more scientific findings were developed. It provides a valuable reference for further exploration of the relationship between teacher support and learning engagement in a technology-involved environment. In terms of practice, scientific guidance should be provided for secondary school teachers on how to improve students’ learning engagement in a technology-infused teaching and learning environment.

The current research has several limitations. First, it used a cross-sectional research design. The use of survey data in this study resulted in our inability to draw causal interpretations about the associations between the variables. This indicates that future research are needed to explore longitudinally the links between perceived teacher support, technology acceptance, learning motivation, and learning engagement, and may assess causal relationships in these directions. Second, there were no differences between schools in this study, but this may because of the fact that the schools studied in this research were all public schools having a small sample size. Therefore, in future studies, it is useful to involve more schools and use a multi-level model for analysis. Third, this study sampled only a subset of middle school students in eastern cities in China. It might be difficult to reflect the correlational link between perceived teacher support and learning engagement among middle school students in all areas of China. In addition, since the research relied on self-reported data acquired only from students, this may contribute to common methodological discrepancies and social expectation biases. Future studies can examine the relationship between teacher support and students’ learning engagement by collecting data from many respondents, including parents, teachers, and students.

## Data availability statement

The original contributions presented in this study are included in the article/supplementary material, further inquiries can be directed to the corresponding author.

## Ethics statement

The studies involving human participants were reviewed and approved by the Ethics Committee of the Jing Hengyi School of Education, Hangzhou Normal University, the Ethical Approval ID is 2022010. The study was conducted according to the guidelines of the Declaration of Helsinki. Written informed consent to participate in this study was provided by the participants’ legal guardian/next of kin.

## Author contributions

FA and JY: conceptualization. JY: methodology and formal analysis. JY and LX: software and data curation. FA: investigation, resources, writing—review and editing, visualization, supervision, project administration, and funding acquisition. All authors have writing—original draft preparation, validation, read, and agreed to the published version of the manuscript.
